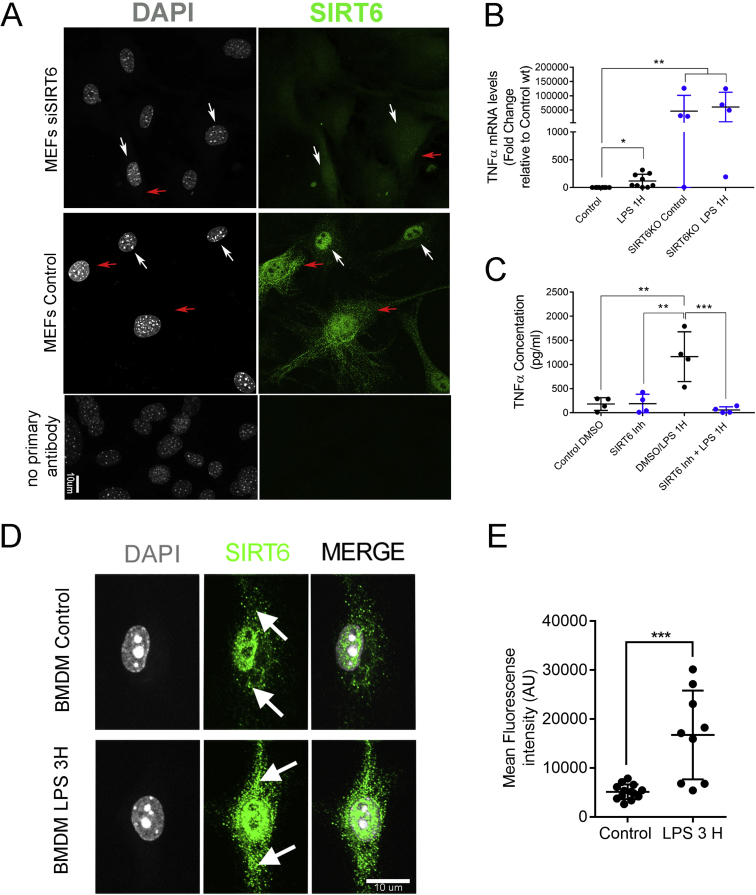# SIRT6 stabilization and cytoplasmic localization in macrophages regulates acute and chronic inflammation in mice

**DOI:** 10.1016/j.jbc.2022.101711

**Published:** 2022-02-09

**Authors:** Mariana Bresque, Karina Cal, Valentina Pérez-Torrado, Laura Colman, Jorge Rodríguez-Duarte, Cecilia Vilaseca, Leonardo Santos, María Pía Garat, Santiago Ruiz, Frances Evans, Rosina Dapueto, Paola Contreras, Aldo Calliari, Carlos Escande

**Affiliations:** 1 Laboratory of Metabolic Diseases and Aging, INDICYO Program, Institut Pasteur Montevideo, Montevideo, Uruguay; 2 Laboratory of Vascular Biology and Drug Development, INDICYO Program, Institut Pasteur Montevideo, Montevideo, Uruguay; 3 Departamento de Biociencias, Facultad de Veterinaria, Universidad de la República (UdelaR), Montevideo, Uruguay; 4 Departamento de Inmunobiología, Facultad de Medicina, Universidad de la República (UdelaR), Montevideo, Uruguay; 5 Área Inmunología, Departamento de Biociencias, Facultad de Química, Universidad de la República (UdelaR), Montevideo, Uruguay; 6 Departamento de Química Orgánica, Facultad de Química, Universidad de la República (UdelaR), Montevideo, Uruguay; 7 Departamento de Fisiología, Facultad de Medicina, Universidad de la República (UdelaR), Montevideo, Uruguay; 8 Departamento de Histología y Embriología, Facultad de Medicina, Universidad de la República (UdelaR), Montevideo, Uruguay; 9 Laboratory of Neuroinflammation and Gene Therapy, Institut Pasteur Montevideo, Montevideo, Uruguay

**Keywords:** sirtuin, inflammation, TNFα, macrophages, obesity, BMDM, bone marrow–derived macrophage, CHIP, carboxyl terminus of Hsp70-interacting protein, CHX, cycloheximide, Csf1r, colony-stimulating factor receptor 1, DIO, diet-induced obesity, DMEM, Dulbecco's modified Eagle's medium, DMSO, dimethyl sulfoxide, EAE, experimental autoimmune encephalomyelitis, ER, endoplasmic reticulum, FBS, fetal bovine serum, LPS, lipopolysaccharide, MEF, mouse embryonic fibroblast, RT, room temperature, SIRT, sirtuin, TCRβ, T-cell receptor beta, TNFα, tumor necrosis factor alpha, WD, Western diet

## Abstract

Acute and chronic inflammations are key homeostatic events in health and disease. Sirtuins (SIRTs), a family of NAD-dependent protein deacylases, play a pivotal role in the regulation of these inflammatory responses. Indeed, SIRTs have anti-inflammatory effects through a myriad of signaling cascades, including histone deacetylation and gene silencing, p65/RelA deacetylation and inactivation, and nucleotide‑binding oligomerization domain, leucine rich repeat, and pyrin domain‑containing protein 3 inflammasome inhibition. Nevertheless, recent findings show that SIRTs, specifically SIRT6, are also necessary for mounting an active inflammatory response in macrophages. SIRT6 has been shown to positively regulate tumor necrosis factor alpha (TNFα) secretion by demyristoylating pro-TNFα in the cytoplasm. However, how SIRT6, a nuclear chromatin-binding protein, fulfills this function in the cytoplasm is currently unknown. Herein, we show by Western blot and immunofluorescence that in macrophages and fibroblasts there is a subpopulation of SIRT6 that is highly unstable and quickly degraded *via* the proteasome. Upon lipopolysaccharide stimulation in Raw 264.7, bone marrow, and peritoneal macrophages, this population of SIRT6 is rapidly stabilized and localizes in the cytoplasm, specifically in the vicinity of the endoplasmic reticulum, promoting TNFα secretion. Furthermore, we also found that acute SIRT6 inhibition dampens TNFα secretion both *in vitro* and *in vivo*, decreasing lipopolysaccharide-induced septic shock. Finally, we tested SIRT6 relevance in systemic inflammation using an obesity-induced chronic inflammatory *in vivo* model, where TNFα plays a key role, and we show that short-term genetic deletion of SIRT6 in macrophages of obese mice ameliorated systemic inflammation and hyperglycemia, suggesting that SIRT6 plays an active role in inflammation-mediated glucose intolerance during obesity.

Sirtuins (SIRTs) are NAD^+^-dependent deacylases that display key regulatory functions in metabolism regulation, cancer, aging, and inflammation (1, 2). In mammals, the family of SIRTs includes seven members (SIRT1–7), with different functions and specific subcellular localizations. SIRT1, SIRT6, and SIRT7 are mainly nuclear proteins; SIRT3–5 localize in the mitochondria, whereas SIRT2 is mainly a cytoplasmic SIRT (3). For a long time, it was believed that the distinct subcellular localization was a signature function for each SIRT. However, accumulated evidence has proven that indeed some SIRTs can shuttle among different cellular compartments depending on specific cues. This is well established for SIRT1 (4), SIRT2 (5), and SIRT3 (6). Despite being traditionally seen as an exclusively nuclear protein, recent evidence suggests that SIRT6 also has cytoplasmic functions (7, 8, 9, 10).

In the nucleus, SIRT6 plays determinant roles in metabolism, genomic stability and aging, and cancer (11). SIRT6 functions mainly as a histone deacetylase (12) and by that means as a regulator of gene expression and genomic stability (11). SIRT6 has poor NAD^+^-dependent deacetylating activity *in vitro*, although this activity is potentiated in the presence of purified and assembled nucleosomes (13). Interestingly, the SIRT6 catalytic pocket accommodates long-chain fatty acids, which stimulate the deacetylase activity (14). More recently, we have shown that long-chain nitro fatty acids potentiate SIRT6 deacetylase activity even in the presence of long-chain fatty acids (15), suggesting that fatty acids are important players in the regulation of SIRT6. Notably, recent findings show that in cultured macrophages and mouse embryonic fibroblasts (MEFs), SIRT6 efficiently removes fatty acid groups from proteins. In fact, it was shown that SIRT6 removes myristoyl groups from pro–tumor necrosis factor alpha (TNFα) in the endoplasmic reticulum (ER) during lipopolysaccharide (LPS)-mediated acute inflammatory response (7, 8). Deletion of SIRT6 impairs TNFα secretion, targeting the protein to lysosomal degradation (7, 8). How SIRT6 localizes to the cytoplasm during this acute inflammatory response is currently unknown. Importantly, although the cytoplasmic localization of SIRT6 is linked to a proinflammatory function, its role in the nucleus is more complex. It was shown that SIRT6 silences NFκB-dependent transcription (16). On the contrary, in pancreatic cancer cells, SIRT6 overexpression increases Ca^2+^ influx indirectly, leading to nuclear factor of activated T cell–dependent TNFα and interleukin 8 induction (9). This suggests that the role of SIRT6 during inflammation is complex and probably under tight spatiotemporal regulation, although the evidence in this sense is scarce. Even more, whether SIRT6 regulates TNFα secretion *in vivo* during inflammation is not known. The possible implications of this regulation go beyond LPS-mediated acute inflammation, since macrophage-derived TNFα is also a driving force for chronic inflammation, glucose intolerance, and tissue damage during obesity (17, 18).

Herein, we show that in macrophages and fibroblasts, LPS treatment promotes a rapid increase in SIRT6 expression that is independent of transcription. Inhibition of the ubiquitin proteasome and blocking of protein synthesis revealed that there is a subpopulation of SIRT6 that is highly unstable. Upon LPS stimulation, SIRT6 is rapidly stabilized in the cytoplasm, locates to the ER, and promotes TNFα secretion. Acute SIRT6 inhibition dampened TNFα secretion both *in vitro* and *in vivo*, showing that SIRT6 is a positive regulator of TNFα secretion *in vivo*. In fact, acute SIRT6 inhibition ameliorated LPS-dependent septic shock. Finally, we show that time-controlled genetic deletion of SIRT6 in macrophages of obese mice decreased obesity-dependent TNFα secretion and ameliorated systemic inflammation and hyperglycemia, suggesting that SIRT6 plays an active role in inflammation-mediated glucose intolerance after the onset of obesity.

## Results

### SIRT6 is rapidly upregulated in response to LPS in macrophages

**Figure 1:**
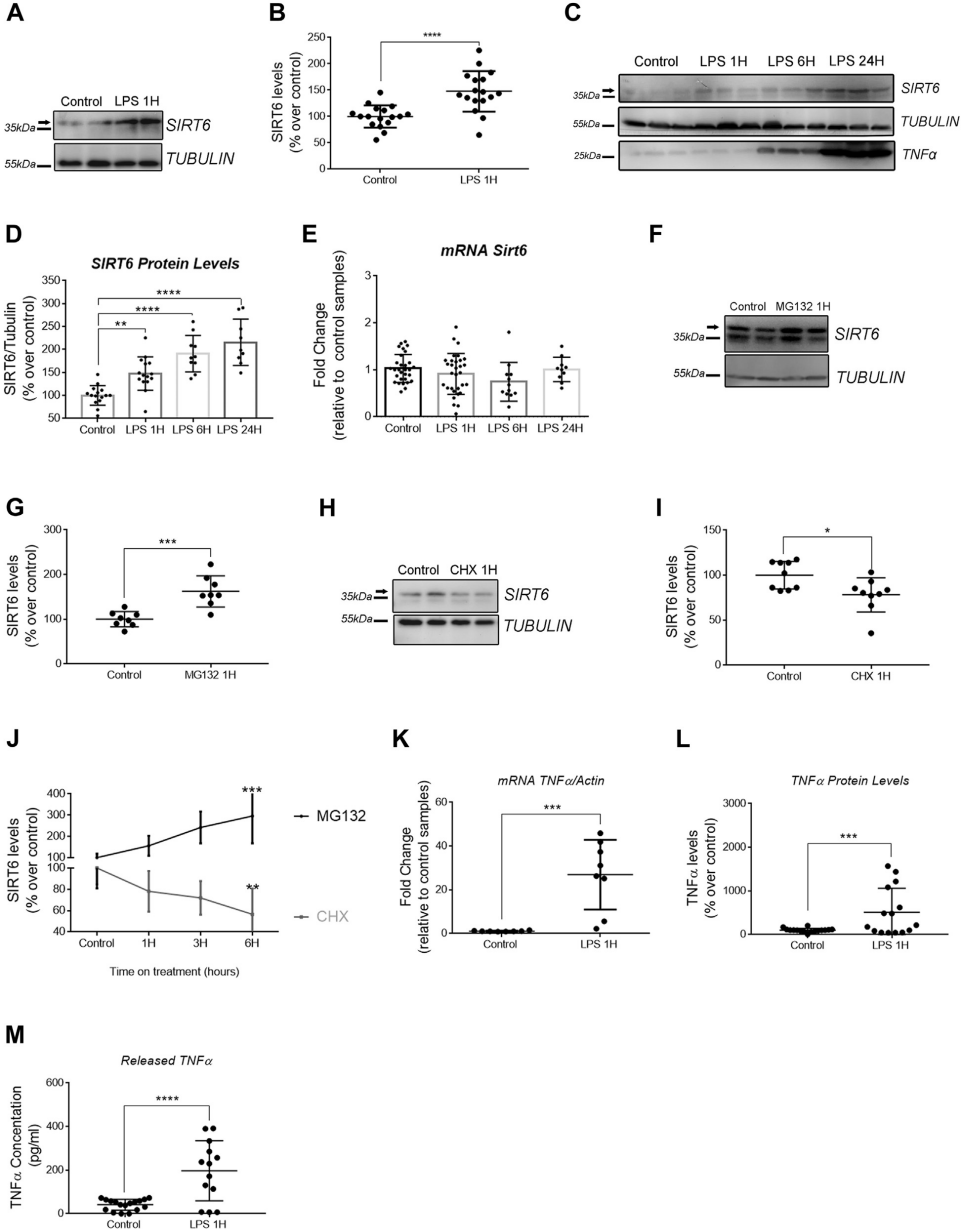
**SIRT6 is upregulated by protein stabilization in response to LPS in macrophages.***A*, representative Western blot (WB) of SIRT6 protein levels in Raw 264.7 cells exposed to LPS (200 ng/ml) for 1 h. Each condition is shown in duplicates. *B*, densitometry analysis of SIRT6 in the conditions described in *A*. *C*, representative WB of SIRT6 protein levels in Raw 264.7 cells exposed to LPS (200 ng/ml) for 1, 6, and 24 h. Each condition is shown in triplicates. *D*, densitometry analysis of SIRT6 as described in *C*. *E*, *Sirt6* mRNA levels in Raw 264.7 cells exposed to LPS (200 ng/ml) for 1, 6, and 24 h. *F* and *G*, representative WB and densitometry analysis of SIRT6 protein levels in Raw 264.7 cells exposed to MG132 for 1 h. *H* and *I*, representative WB and densitometry analysis of SIRT6 protein levels in Raw 264.7 cells exposed to cycloheximide (CHX, 100 μg/ml) for 1 h. *J*, densitometry of SIRT6 levels relative to control samples in Raw 264.7 cells exposed to MG132 (10 μM) for 1, 3, and 6 h (*black line*) and CHX (1 μg/ml) for 1, 3, and 6 h (*gray line*) treatments. *K*, *Tnfα* mRNA levels in Raw 264.7 cells exposed to LPS (200 ng/ml) for 1 h. *L*, densitometry readings of TNFα relative to tubulin of WB showed in *A* and its replicates. *M*, released TNFα levels measured by ELISA in the supernatant of Raw 264.7 cells exposed to LPS (200 ng/ml) for 1 h. Data represent mean ± SD, ∗*p* < 0.05, ∗∗*p* < 0.01, ∗∗∗*p* < 0.001, and ∗∗∗∗*p* < 00.001. All experiments were repeated four times. LPS, lipopolysaccharide; SIRT6, sirtuin 6; TNFα, tumor necrosis factor alpha.

**Supplemental Figure S1:**
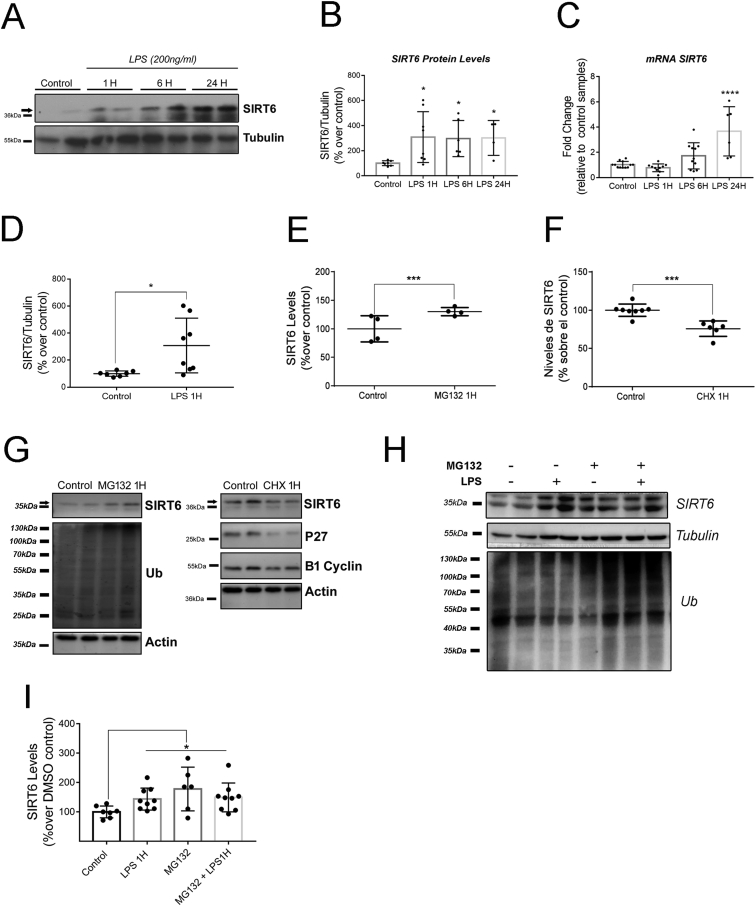


In order to gain insight into how SIRT6 is regulated during acute inflammatory response, we treated Raw 264.7 macrophages with LPS (200 ng/ml) for different periods. We found that LPS promoted a fast increase in SIRT6 protein levels that became significant as early as 1 h after stimulation (Fig. 1, *A* and *B*). This SIRT6 accumulation was time dependent and continued to rise for up to 24 h after LPS stimulation (Fig. 1, *C* and *D*). Surprisingly, the increase in protein expression was not preceded by changes in mRNA (Fig. 1*E*). Based on these results, we sought to determine if SIRT6 could be subject to post-transcriptional regulation, leading to a fast upregulation during inflammatory response. For that, we treated Raw 264.7 cells with LPS with the proteasome inhibitor MG132 and with the protein synthesis inhibitor cycloheximide (CHX) for a short period (1 h). We found that MG132 led to a fast and comparable increase in SIRT6 protein (Fig. 1, *F* and *G*). On the contrary, inhibition of protein synthesis with CHX generated a time-dependent decrease in SIRT6 protein levels (Fig. 1, *H* and *I*). Time response experiments with CHX and MG132 showed that in macrophages SIRT6 has a short half-life (∼3 h, Fig. 1*J*). The fast upregulation of SIRT6 protein levels in response to LPS treatment was coincidental with the increase in TNFα synthesis and secretion (Fig. 1, *K*–*M*). Similar results were obtained using MEFs. In these cells, LPS led to a rapid increase in SIRT6 protein levels independently of mRNA, and this SIRT6 protein increase was sensitive to MG132 and CHX. Furthermore, incubation of cells with combination of LPS and MG132 produced similar changes as either stimuli alone, suggesting that the same pool of SIRT6 is being regulated by both compounds (Fig. S1).

### Upon LPS stimulation, stabilized SIRT6 localizes in the nucleus and cytoplasm

**Figure 2:**
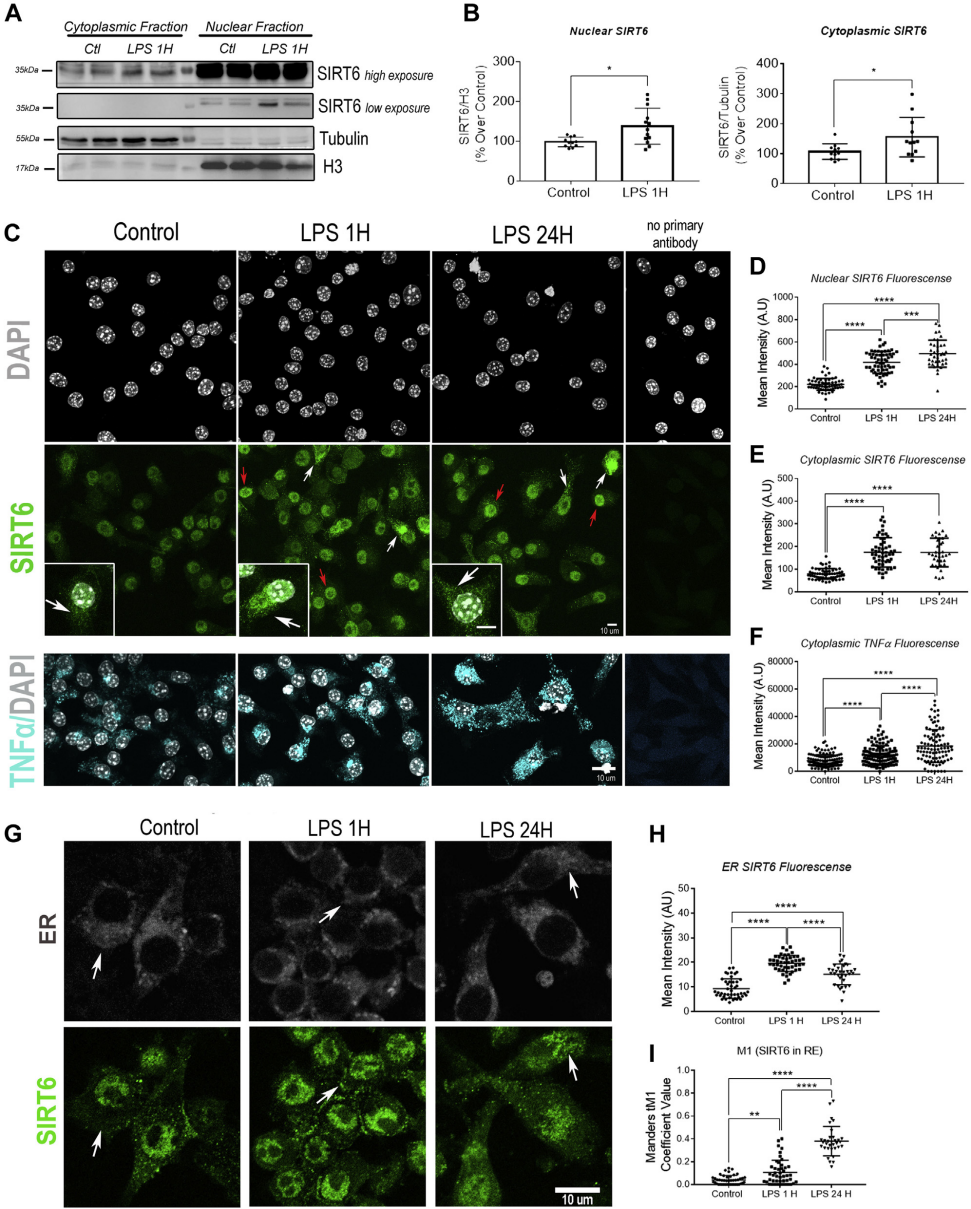
**LPS promotes SIRT6 cytoplasmic and ER accumulation in macrophages.***A*, representative Western blot of SIRT6 in cytoplasmic and nuclear fractions from Raw 264.7 cells incubated with LPS (200 ng/ml) for 1 h. Tubulin and H3 were used as markers for cytoplasmic and nuclear fractions, respectively. *B*, densitometry analysis of SIRT6 expression in the conditions described in *A*. *C*, representative immunofluorescence (IF) confocal images of Raw 264.7 cells incubated with LPS (200 ng/ml) for 1 and 24 h. Cells were labeled with DAPI (*gray*), SIRT6 (*green*), and TNFα (*cyan*). *Right panel* shows IF control with no primary antibody. The *insets* represent the magnification of the framed nuclei. *White arrows* indicate cytoplasmic SIRT6 accumulations, and *red arrows* indicate nuclear SIRT6. The scale bar represents 10 μm. *D* and *E*, mean fluorescence intensity quantification in nuclear (*D*) and cytoplasmic (*E*) SIRT6 signals in response to LPS treatments. *F*, mean fluorescence intensity quantification of TNFα in the cytoplasm. *G*, representative IF of Raw 264.7 cells exposed to LPS (200 ng/ml) for 1 and 24 h staining SIRT6 (*green*) and ER (*white*). *White arrows* indicate SIRT6 accumulations to the ER. The scale bar represents 10 μm. *H*, SIRT6 mean fluorescence intensity quantification in ER regions. *I*, Manders M1 coefficient value in Raw 264.7 cells exposed to LPS (200 ng/ml) for 1 and 24 h. Data represent mean ± SD, ∗*p* < 0.05, ∗∗*p* < 0.01, ∗∗∗*p* < 0.001, and ∗∗∗∗*p* < 00.001. All experiments were repeated four times. DAPI, 4′,6-diamidino-2-phenylindole; ER, endoplasmic reticulum; LPS, lipopolysaccharide; SIRT6, sirtuin 6; TNFα, tumor necrosis factor alpha.

Next, we determined the subcellular localization of SIRT6 during LPS-induced macrophage activation. Subcellular fractionation showed that LPS promoted a clear upregulation of SIRT6 in the cytoplasm and also to some extent in the nucleus (Fig. 2, *A* and *B*). Immunofluorescence analysis and quantitation on Raw 264.7 cells and in bone marrow–derived macrophages (BMDMs) showed that LPS led to an accumulation in SIRT6 both in the nucleus and cytoplasm (Figs. 2, *C*–*E* and S2). The specificity of the cytoplasmic signaling of SIRT6 was validated by siRNA-mediated SIRT6 knockdown (Fig. S2). The increase in SIRT6 cytoplasmic signal was statistically significant 1 h after treatment and remained high for up to 24 h (Fig. 2*E*). Since it has been proposed that cytoplasmic SIRT6 is necessary for TNFα secretion (7, 8), we sought to determine if rapid cytoplasmic SIRT6 upregulation could play a role in this pathway. TNFα is synthesized and secreted by the classical secretory pathway. Once in the ER, the cytoplasmic tail of TNFα has to be demyristoylated by SIRT6 in order to avoid being targeted to lysosomes and instead traffic to the cell surface (8). We measured TNFα in the cells in response to LPS, and similar to SIRT6, we found an increase in its cytoplasmic staining 1 h after LPS treatment, remaining high even 24 h later (Fig. 2*F*). Next, we aimed to determine if the increase in cytoplasmic SIRT6 corresponded with an ER accumulation of the protein. We labeled the ER with ER-Tracker Red Dye (Fig. 2*G*) and then measured the amount of SIRT6 in the ER area. We found that upon LPS treatment, SIRT6 readily accumulated in the ER area within 1 h of LPS treatment (Fig. 2*H*), coincidental with TNFα increase (Fig. 2*F*). Furthermore, we measured the Mander's overlap coefficient and found that LPS treatment led to an increase in the localization of SIRT6 into the ER (Fig. 2*I*).

### Acute SIRT6 inhibition decreases TNFα secretion without affecting its intracellular expression

**Figure 3:**
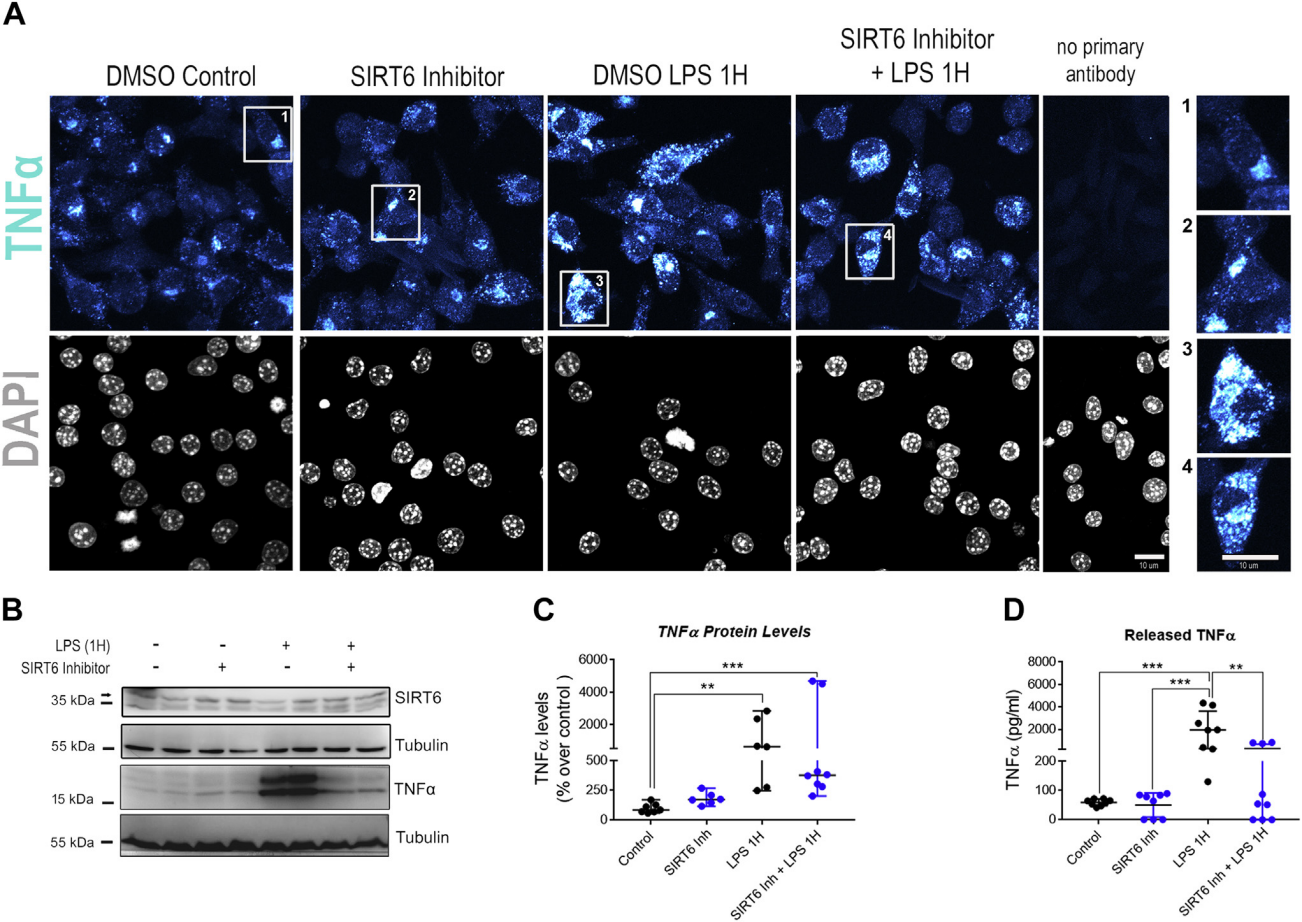
**Acute SIRT6 inhibition *in vitro* impairs TNFα release in LPS-treated macrophages.***A*, representative IF confocal images of Raw 264.7 cells incubated with LPS (200 ng/ml) and SIRT6 inhibitor (compound 1, 200 μM) for 1 h. Cells were stained for DAPI (*gray*) and TNFα (*cyan*). *Numbered squares* correspond to magnifications in the *right panel*. The scale bar represents 10 μm. *B*, representative Western blot for TNFα in Raw 264.7 cells exposed to LPS (200 ng/ml) and SIRT6 inhibitor (compound 1, 200 μM) for 1 h. *C*, densitometry measurements of intracellular TNFα expression in the experiments described in *B*. *D*, levels of released TNFα to the cell media measured by ELISA in the supernatant of Raw 264.7 cells incubated with LPS (200 ng/ml) and the SIRT6 inhibitor (compound 1, 200 μM) for 1 h. Data represent median ± 95% confidence interval (*C*) and mean ± SD (*D*), ∗*p* < 0.05, ∗∗*p* < 0.01, ∗∗∗*p* < 0.001, and ∗∗∗∗*p* < 00.001. All experiments were repeated four times. DAPI, 4′,6-diamidino-2-phenylindole; IF, immunofluorescence; LPS, lipopolysaccharide; SIRT6, sirtuin 6; TNFα, tumor necrosis factor alpha.

In order to determine if SIRT6 stabilization and cytoplasmic localization was directly linked to TNFα secretion, we treated cells with the recently described (19) SIRT6 inhibitor 2,4-dioxo-*N*-[4-(pyridin-3-yloxy)phenyl]-1,2,3,4-tetrahydroquinazoline-6-sulfonamide (compound 1). Since chronic SIRT6 deletion promotes TNFα transcription (Fig. S2), we sought to minimize this effect by treating cells acutely with compound 1, together with LPS stimulation. LPS-dependent intracellular TNFα protein levels were not affected by the inhibitor, as immunofluorescence staining showed similar staining and distribution of intracellular TNFα in response to LPS in the presence or the absence of compound 1 (Fig. 3*A*). A basal TNFα signal could be detected in some cells, an effect that could be attributed to the dimethyl sulfoxide (DMSO) treatment, since it was also seen in control cells (Fig. 3*A*). We further confirmed these results by Western blot, where we found that the SIRT6 inhibitor did not significantly affect intracellular TNFα levels in response to LPS (Fig. 3, *B* and *C*). However, when we measured the amount of TNFα secreted to the cellular medium, we found that inhibition of SIRT6 significantly reduced the LPS-mediated TNFα secretion (Fig. 3*D*), supporting our hypothesis that rapid upregulation of cytoplasmic SIRT6 accumulation and localization to the ER in response to LPS is necessary of TNFα secretion. Similar results for TNFα secretion were obtained in MEFs (Fig. S2).

### Acute SIRT6 inhibition *in vivo* inhibits LPS-induced TNFα secretion and reduces septic shock

**Figure 4:**
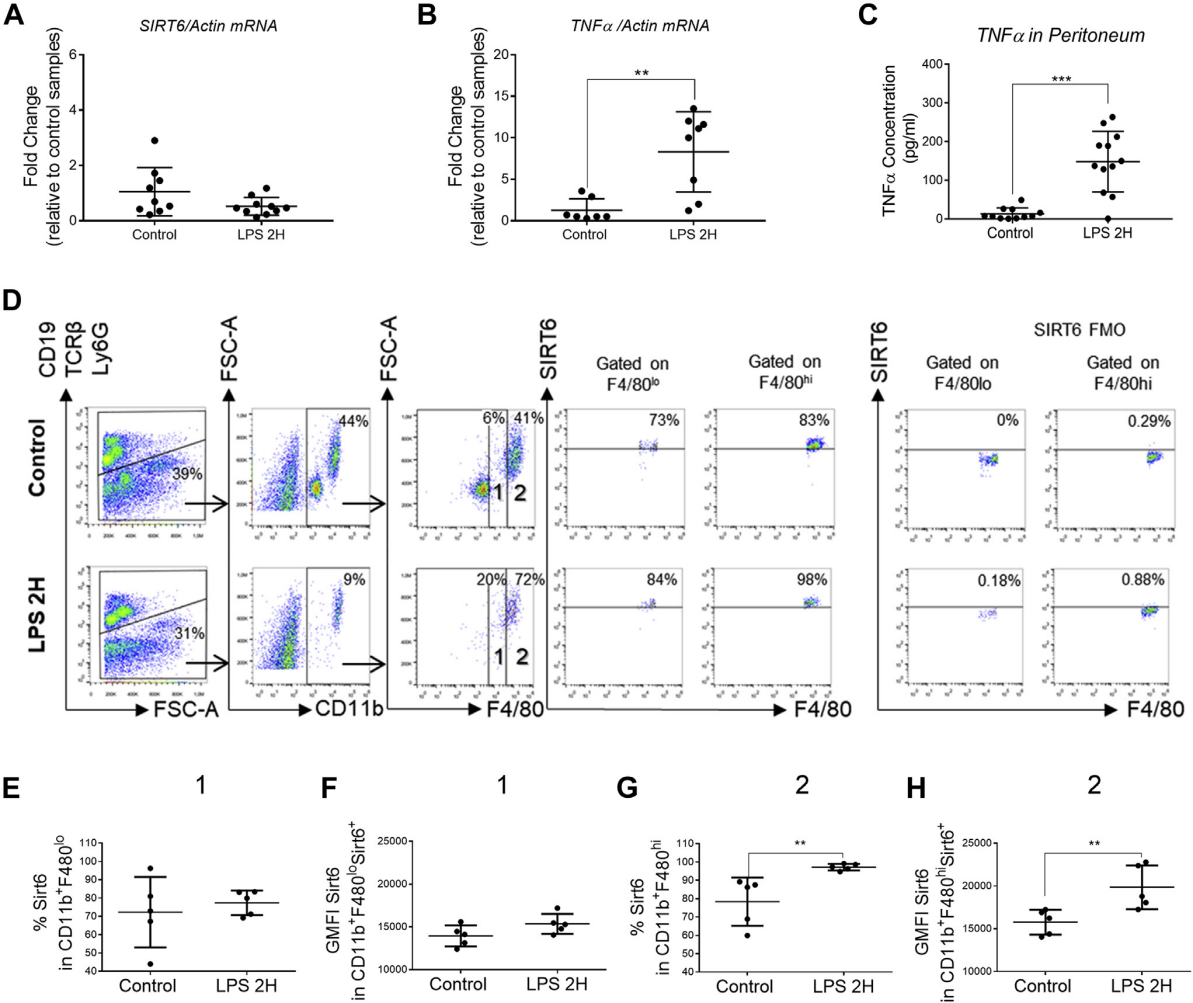
**LPS administration to mice promotes rapid SIRT6 upregulation in resident peritoneal macrophages.***A* and *B*, SIRT6 and TNFα mRNA levels in peritoneal cavity cells obtained after LPS injection (10 mg/kg) for 2 h. *C*, released TNFα levels measured by ELISA in the peritoneal lavage of mice after LPS injection (10 mg/kg) for 2 h. *D*–*H*, flow cytometry analysis of SIRT6 protein levels in CD11b^+^F4/80^lo^ (recruited) and CD11b^+^F4/80^hi^ (resident) macrophages within peritoneal cavity cells obtained after LPS injection. *D*, representative dot plots showing the gating strategy used for the identification of CD11b^+^F4/80^lo^ and CD11b^+^F4/80^hi^ macrophages and SIRT6 analysis in these cells; SIRT6 FMOs were used for the determination of SIRT6-positive cells in each condition. *E*–*H*, quantitation of SIRT6 protein levels in CD11b^+^F4/80^lo^ and CD11b^+^F4/80^h^^i^ macrophages, shown as percentage of SIRT6-positive cells (*E* and *G*) and SIRT6 geometric mean fluorescence intensity (GMFI) in SIRT6-positive cells within these populations (*F* and *H*). Data represent mean ± SD, ∗*p* < 0.05, ∗∗*p* < 0.01, ∗∗∗*p* < 0.001, and ∗∗∗∗*p* < 00.001 (n = 5). FMO, Fluorescence Minus One; LPS, lipopolysaccharide; SIRT6, sirtuin 6; TNFα, tumor necrosis factor alpha.

**Figure 5:**
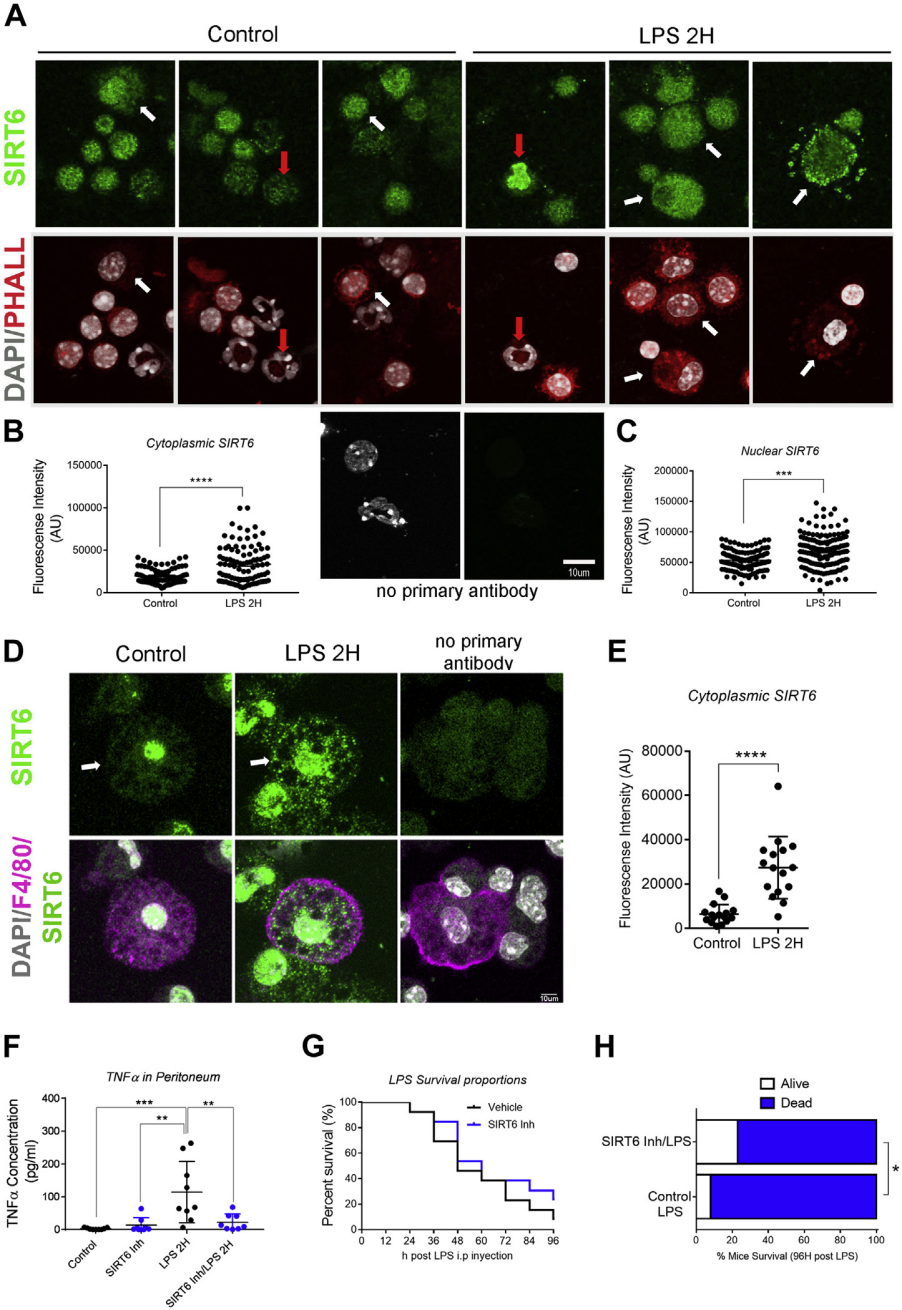
***In vivo* pharmacological SIRT6 inhibition decreases TNF**α **secretion after LPS stimulation and reduces LPS-dependent mortality.***A*, representative confocal immunofluorescence images of cells obtained from the peritoneal cavity after LPS injection, stained with DAPI (*gray*), phalloidin (*red*), and SIRT6 (*green*). *White arrows* indicate cytoplasmic SIRT6 accumulations, and *red arrows* indicate cells with high SIRT6 signals. The scale bar represents 10 μm. *B* and *C*, SIRT6 mean fluorescence intensity quantification in nuclear (*B*) and corresponding cytoplasmic fractions (*C*) obtained from peritoneal cavity cells after LPS injection in mice. *D* and *E*, representative confocal immunofluorescence images of thioglycollate-elicited macrophages in response to LPS treatment (10 mg/kg, 2 h). F4/80 (*magenta*) was used as a marker for macrophages, and SIRT6 (*green*) was quantified in macrophage cytoplasm (*E*). *White arrows* indicate cytoplasmic SIRT6 accumulations. The scale bar represents 10 μm. *F*, released TNFα levels measured by ELISA in the peritoneal cavity lavage of mice exposed to LPS with or combination of LPS and SIRT6 inhibitor (compound 1, 30 mg/kg) (n = 7). *G*, Kaplan–Meier survival curve after treating mice with LPS (20 mg/kg) or LPS + SIRT6 inhibitor (compound 1, 30 mg/kg). *H*, contingency graph showing survival after an LPS lethal injection (20 mg/kg) with or without the administration of SIRT6 inhibitor (compound 1, 30 mg/kg) (n = 14 per experimental group). Data represent mean ± SD, ∗*p* < 0.05, ∗∗*p* < 0.01, ∗∗∗*p* < 0.001, and ∗∗∗∗*p* < 00.001. DAPI, 4′,6-diamidino-2-phenylindole; LPS, lipopolysaccharide; SIRT6, sirtuin 6; TNFα, tumor necrosis factor alpha.

The effects of SIRT6 inhibition in systemic inflammation are varied and sometimes opposite. While genetic, chronic SIRT6 inhibition in macrophages promotes inflammation and insulin resistance (20), pharmacological inhibition of SIRT6 ameliorates glucose intolerance during diet-induced obesity (DIO) (19). In the same line, recent findings in an experimental autoimmune encephalomyelitis (EAE) model show that acute pharmacological SIRT6 inhibition decreases TNFα secretion (21). Since nuclear and cytoplasmic SIRT6 seem to play opposite roles in relation to TNFα secretion and inflammation, dissecting *in vivo* effects may need to consider the time frame during which SIRT6 activity is predominant. First, we sought to determine if SIRT6 was acutely upregulated during inflammation *in vivo*. For this purpose, we treated mice with LPS (10 mg/kg, IP) for 2 h and then isolated the peritoneal cavity cells and lavage fluid. Expression profile from total peritoneal cavity cell fraction showed no changes in SIRT6 mRNA (Fig. 4*A*). As expected, TNFα secretion into peritoneal cavity fluid was significantly upregulated by the treatment with LPS (Fig. 4, *B* and *C*). Next, we analyzed SIRT6 protein levels in macrophages from the peritoneal cavity by flow cytometry (Fig. 4, *D*–*H*). In the case of CD11b^+^F4/80^lo^ (recruited) macrophages, LPS did not affect SIRT6 protein levels, either in the percent of SIRT6-positive cells (Fig. 4*E*) or in SIRT6 fluorescence intensity (measured as geometric mean fluorescence intensity in SIRT6^+^ cells) (Fig. 4*F*). However, when we analyzed CD11b^+^F4/80^hi^ (resident) macrophages, we found that LPS led to a significant increase in both SIRT6-positive cells (Fig. 4*G*) and SIRT6 fluorescence intensity within those cells (Fig. 4*H*). Immunofluorescence analysis of the SIRT6 subcellular localization in the peritoneal cavity cellular fraction showed a significant increase in SIRT6 staining both in the nucleus and in the cytoplasm after LPS treatment (Fig. 5, *A*–*C*). The effect of cytoplasmic SIRT6 accumulation *in vivo* during LPS stimulation was further confirmed in thioglycollate-elicited macrophages (Fig. 5, *D* and *E*). Finally, we investigated if acute SIRT6 inhibition decreased the LPS-dependent inflammatory response and septic shock *in vivo*. We treated mice with LPS (20 mg/kg) or LPS + compound 1 (30 mg/kg) for 2 h and found that SIRT6 inhibition completely blocked acute TNFα secretion *in vivo* (Fig. 5*F*). Consistent with this, SIRT6 inhibition modestly decreased LPS-induced septic shock and mortality (Fig. 5, *G* and *H*) and systemic effect of SIRT6 during the acute inflammatory response.

### Genetic deletion of SIRT6 in macrophages ameliorates systemic inflammation and hyperglycemia in obese mice

**Figure 6:**
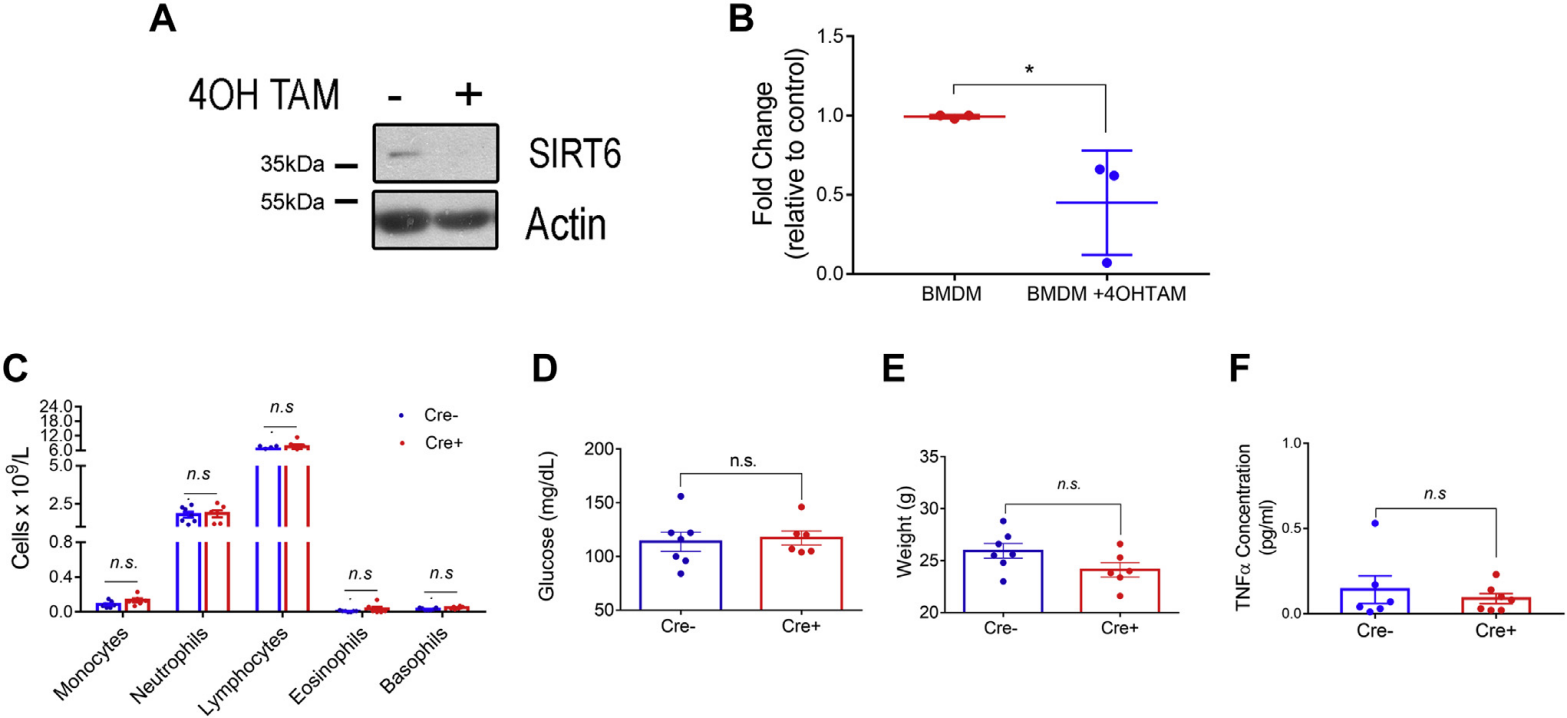
**Tamoxifen-induced SIRT6 deletion in macrophages does not affect systemic inflammation and glucose management in lean mice.***A*, representative Western blot showing the effect of 4-OH-tamoxifen (2 μM) on SIRT6 protein levels in BMDM isolated and cultured from Sirt6^loxP/loxP^;Cre+ mice. *B*, *Sirt6* mRNA levels in BMDM isolated and cultured from Sirt6^loxP/loxP^;Cre+ mice and exposed to 4-OH-tamoxifen. *C*, peripheral blood cell count in Sirt6^loxP/loxP^;Cre+ and Sirt6^loxP/loxP^;Cre− mice after tamoxifen treatment. *D*, basal glycemia of Sirt6^loxP/loxP^;Cre+ and Sirt6^loxP/loxP^;Cre− mice after tamoxifen treatment. *E*, body weight of Sirt6^loxP/loxP^;Cre+ and Sirt6^loxP/loxP^;Cre− mice after tamoxifen treatment. *F*, released TNFα levels measured by ELISA in the peritoneal lavage of Sirt6^loxP/loxP^;Cre+ and Sirt6^loxP/loxP^;Cre− mice after tamoxifen treatment. Data represent mean ± SD, ∗*p* < 0.05, ∗∗*p* < 0.01, ∗∗∗*p* < 0.001, and ∗∗∗∗*p* < 00.001. BMDM, bone marrow–derived macrophage; SIRT6, sirtuin 6; TNFα, tumor necrosis factor alpha.

It is well established that chronic inflammation is a major driving force for the deleterious effects of obesity and has been directly linked to the progression of hyperglycemia, insulin resistance, nonalcoholic fatty liver disease, and atherosclerosis (22). In fact, TNFα has been unequivocally linked to these phenomena (17, 18, 23, 24). To test the possible role of SIRT6 on TNFα secretion after the onset of chronic inflammation during obesity, we developed a genetic mouse model for inducible SIRT6 deletion in macrophage colony-stimulating factor receptor 1 (Csf1r)–positive macrophages. For this purpose, we crossed Sirt6^loxp/loxp^ × Csf1r-Mer-iCre-Mer mice and put them on a C57BL6/J background (Sirt6^loxp/loxp^,Cre+; see methods for detailed mouse generation and background homogenization). The effectiveness of tamoxifen-induced SIRT6 deletion in macrophages was first determined in BMDM *in vitro* (Fig. 6, *A* and *B*). Next, we treated Sirt6^loxp/loxp^;Cre− and Sirt6^loxp/loxp^;Cre+ with tamoxifen (50 mg/kg/day) for 1 week before analyzing circulating monocytes, neutrophils, basophils, eosinophils, and lymphocytes (Fig. 6*C*) as well as basal glucose levels and body weight (Fig. 6, *D* and *E*). We found no significant difference among genotypes treated with tamoxifen when the mice were on normal chow. Furthermore, we measured secreted TNFα in the peritoneal cavity and found no differences among genotypes (Fig. 6*F*). In summary, tamoxifen treatment led to a significant decrease in SIRT6 expression in macrophages, without affecting inflammation or glucose management when mice were fed on normal chow.

**Figure 7:**
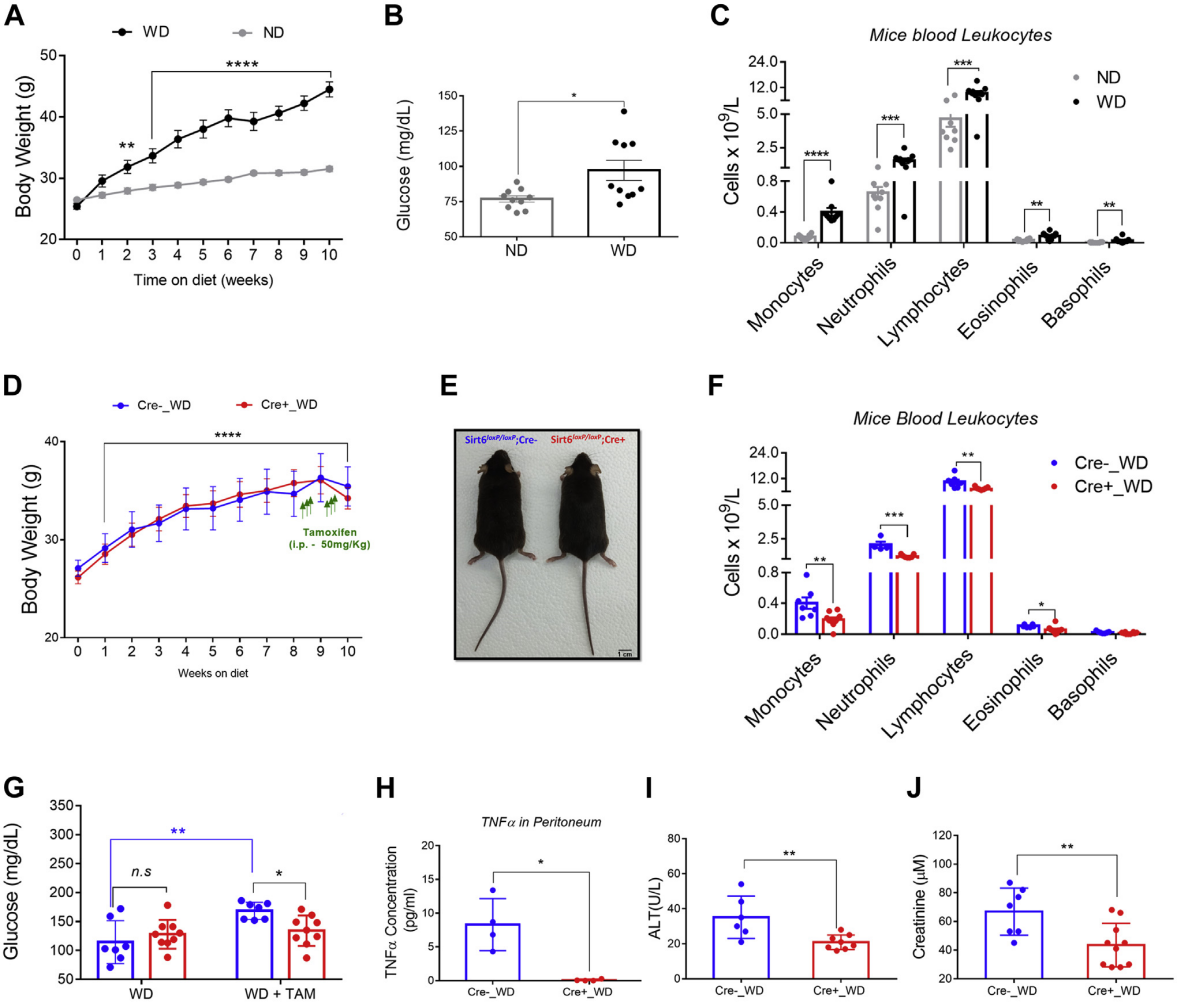
**Tamoxifen-induced SIRT6 deletion in macrophages after the onset of obesity ameliorates systemic inflammation and hyperglycemia.***A*, body weight gain in mice fed with normal chow diet (ND, *gray line*) and Western diet (WD, *black line*) (n = 10). *B*, basal glycemia in lean (ND) and obese (WD) mice 10 weeks after the onset of the treatment. *C*, peripheral blood cell count in mice in ND and WD 10 weeks after the onset of the treatment. *D*, body weight gain of Sirt6^loxP/loxP^;Cre− and Sirt6^loxP/loxP^;Cre+ mice fed with WD (n = 7). *Green arrows* indicate sequential and daily tamoxifen (50 mg/kg, subcutaneously [SC]) injections. *E*, representative picture of Sirt6^loxP/loxP^;Cre− and Sirt6^loxP/loxP^;Cre+ mice at the end of the treatment (10 weeks of WD + tamoxifen). *F*, hemogram of peripheral blood cells of Sirt6^loxP/loxP^;Cre− and Sirt6^loxP/loxP^;Cre+ mice in WD and after the treatment with tamoxifen (50 mg/kg, SC). *G*, fasting glucose levels in mice in WD before and after tamoxifen (50 mg/kg, SC) treatment. *H*, released TNFα levels measured by ELISA in the peritoneal lavage of Sirt6^loxP/loxP^;Cre− and Sirt6^loxP/loxP^;Cre+ under WD + 2 weeks of tamoxifen (50 mg/kg, SC) sequential injections. *I* and *J*, alanine aminotransferase (ALT) and creatinine levels from mice. *Blue lines* show Sirt6^loxP/loxP^;Cre− and *red lines* show Sirt6^loxP/loxP^;Cre+ mice in WD. SIRT6, sirtuin 6; TNFα, tumor necrosis factor alpha.

Next, we used the extensively analyzed DIO model of systemic inflammation and glucose intolerance in C57BL6/J mice as previously described (25, 26). Mice were fed with a Western-style diet for 10 weeks. During that period, mice consistently became obese (Fig. 7*A*) as well as developed hyperglycemia (Fig. 7*B*) and systemic inflammation (Fig. 7*C*). Based on our results and previous work, we hypothesized that constitutive and time-controlled SIRT6 deletion or inhibition can lead to different outcomes in terms of systemic inflammation. Taking this into consideration, we decided to induce SIRT6 deletion in macrophages for a short period, and after that mice became obese. We put them on Western diet (WD) and followed weight gain for 10 weeks. Sirt6^loxp/loxp^;Cre− and Sirt6^loxp/loxp^;Cre+ mice gained weight in a similar manner (Fig. 7, *D* and *E*). Between weeks 8 to 9 of WD, we treated them with tamoxifen. We found that SIRT6 deletion in macrophages partially protected mice against systemic inflammation (Fig. 7*F*). Consistent with this, we also found a significant protection against hyperglycemia (Fig. 7*G*), strongly suggesting that conditional SIRT6 deletion in macrophages once obesity is established prevents systemic inflammation and hyperglycemia. We measured TNFα levels in plasma, but even in the mice on WD, we could not detect measurable levels of the cytokine. As a proof of principle, we turned to measure TNFα levels in the peritoneal cavity of the obese Sirt6^loxp/loxp^;Cre− and Sirt6^loxp/loxp^;Cre+ mice treated with tamoxifen. We found that Sirt6^loxp/loxp^;Cre− mice had significantly higher levels of secreted TNFα in the peritoneal cavity than Sirt6^loxp/loxp^;Cre + mice (Fig. 7*H*), confirming that SIRT6 deletion in macrophages ameliorates TNFα secretion *in vivo* during obesity. Finally, we measured liver and kidney function and found a mild, although significant, protection against liver and kidney damage (Fig. 7, *I* and *J* and Table S1).

## Discussion

SIRTs play a myriad of protective roles in metabolism, cell cycle regulation and cancer, aging, and inflammation. While SIRTs, especially SIRT1 and SIRT6, seem to have anti-inflammatory functions by silencing NFκB-dependent gene expression in the nucleus (1), recent evidence shows that, at least in macrophages, fibroblasts (7, 8), and dendritic cells (27), SIRT6 can have different, and even opposite, functions in regulating inflammation. How the cell manages SIRT6 activity and localization in order to orchestrate these *a priori* opposite actions is not completely understood. The fact that the proinflammatory and anti-inflammatory actions of SIRT6 occur in different subcellular compartments (cytoplasm and nucleus, respectively) suggests that protein compartmentalization may play a role in this phenomenon. Previous work has shown that during LPS treatment, SIRT6 appears to cosediment with the ER fraction upon subcellular fractionation (7). However, how SIRT6 ends up in the cytoplasm during this response is not established. Also, until now, evidence for the proinflammatory function of SIRT6 through positive regulation of TNFα secretion has been focused on cell culture experiments, with limited experimental *in vivo* support. In this work, we intended to shed light on these questions.

LPS promoted rapid SIRT6 upregulation that was not dependent on transcription but on protein stabilization instead. It has been shown that SIRT6 protein levels and activity is regulated by ubiquitination and proteasome-dependent degradation. In particular, it has been shown that the ubiquitin ligase carboxyl terminus of Hsp70-interacting protein (CHIP) stabilizes SIRT6 and prevents proteasomal degradation by noncanonical ubiquitination (28). On the other hand, ubiquitin-specific peptidase 10 promotes SIRT6 stabilization by preventing ubiquitination and degradation (29). Interestingly, these previous reports show an important difference in the half-life of the protein, going from 1 to 2 h to 24 to 48 h, suggesting that the stability of SIRT6 may be differentially regulated in different cellular types and contexts. Our results *in vivo* are aligned with these reports, since we showed an increased SIRT6 level in CD11b^+^F4/80^hi^ (resident) peritoneal macrophages in response to LPS but not in CD11b^+^F4/80^lo^ (recruited) peritoneal macrophages, suggesting that SIRT6 acute regulation may differ among macrophage subtypes. The ubiquitin ligase CHIP has been linked to LPS-dependent signaling and inflammatory response in macrophages (30), and it also regulates SIRT6 stability in MEFs (28), suggesting that this ubiquitin ligase is a good candidate to mediate LPS-dependent SIRT6 stabilization. SIRT6 protein stabilization by LPS led to clear increase in cytoplasmic and specifically ER localization of the protein. However, we also found a rapid increase in nuclear SIRT6. Whether SIRT6 nuclear accumulation is a result of cytoplasmic SIRT6 stabilization and nuclear leakage, or because of specific nuclear stabilization, remains to be studied. Recent evidence shows that the ubiquitin proteasome machinery is present in the nucleus (31, 32), so a specific nuclear regulation of SIRT6 stability is plausible. It has been recently proposed that SIRT6 can be moved to the cytoplasm by p62 during cadmium-induced cellular toxicity (10). Although we cannot rule out a nuclear–cytoplasmic shuttling of SIRT6 and cytoplasmic accumulation during LPS stimulation, the kinetics of nuclear and cytoplasmic accumulation suggest otherwise. While cytoplasmic SIRT6 upregulation peaked at 1 h post-LPS incubation and remained constant until 24 h later, nuclear SIRT6 continued to accumulate even after 24 h. This supports the notion that upon LPS stimulation, SIRT6 is stabilized in the cytoplasm for the duration of the stimulus, leading to a continuous leak into the nucleus regulated by the nuclear localization signal in SIRT6. Accumulated cytoplasmic SIRT6 showed an enrichment in the ER area, consistent with our hypothesis that SIRT6 stabilization plays a role in TNFα demyristoylation as previously shown (7). In fact, SIRT6 inhibition decreased TNFα secretion without clearly affecting intracellular protein levels, although important variability was observed. This variability could be explained by fast degradation because of SIRT6 inhibition (8). It is important to mention that not all cytoplasmic SIRT6s localized to the ER. Whether the remaining cytoplasmic SIRT6 plays a different role in the cytoplasm, or is just a bystander of the actual regulatory role of the protein in TNFα secretion, could not be sorted out. Nevertheless, clarification of this issue will deserve further investigation.

While it was very well established that SIRT6 plays a pivotal proinflammatory role during TNFα secretion, *in vivo* evidence of this regulation is scarce. Recently, it was shown that pharmacological SIRT6 inhibition in an EAE model delays the onset of the disease through a lower dendritic cell activation and migration, which correlated with decreased TNFα in plasma (21). However, most *in vivo* evidence has ascribed an anti-inflammatory role of SIRT6. Here, we are providing direct evidence that SIRT6 is rapidly upregulated in macrophages *in vivo* during LPS-induced inflammation. In fact, LPS promoted an upregulation of SIRT6 that also localized in the cytoplasm and nucleus. Consistent with this, SIRT6 inhibition decreased LPS-induced TNFα secretion *in vivo*, suggesting that cytoplasmic SIRT6 upregulation in macrophages *in vivo* is also required for this response. Interestingly, LPS-induced SIRT6 upregulation was restricted to CD11b^+^F4/80^hi^ resident macrophages, suggesting that in different subsets of macrophages, SIRT6 regulation may differ. In addition, we cannot rule out the possibility that SIRT6 inhibition impairs macrophage migration to the peritoneum, as has been described for BxPC-3 cells and dendritic cells (9, 21).

Our findings extend the role of SIRT6 in the regulation of TNFα-mediated inflammation to chronic inflammation and glucose management during obesity. This is not the first time that SIRT6 inhibition is linked to a better outcome in glucose management. Recent work has shown that pharmacological SIRT6 inhibition ameliorates glucose intolerance and tissue damage during obesity, although this effect was ascribed to nuclear control of the expression of glycolytic genes. Whether TNFα-related inflammation was contributing to this phenotype was not studied (19). In the same line, as mentioned previously, pharmacological SIRT6 inhibition in an experimental model of EAE ameliorated progression of the disease, including diminished TNFα levels *in vivo* (21). However, other evidence points in the opposite direction. Constitutive SIRT6 deletion in myeloid cells promotes inflammation and tissue damage during obesity by promoting macrophage polarization toward an M1 phenotype (20), clearly showing that constitutive SIRT6 deletion in myeloid cells has deleterious effects during obesity and inflammation. Nevertheless, it is not clear to what extent this phenotype is influenced by constitutive SIRT6 deletion in the whole myeloid population, and in particular, what is the participation of nonmacrophage myeloid cell types. In our mice model, we generated a new macrophage-specific Sirt6 KO mice, that can be temporally activated by tamoxifen. Macrophage Sirt6 deletion was corroborated in BMDM under tamoxifen addition in culture. No differences were found in control and transgenic animals in normal diet as well as in WD before tamoxifen injections, suggesting nonspecific Cre effects in parameters measured. Nonetheless, Cre nonspecific effects have been described for stimulator of interferon gene antiviral pathway activation and the induction of type 1 interferon (33), so future work should also consider this possibility. We found that SIRT6 downregulation in macrophages after the onset of obesity, decreased systemic inflammation and TNFα secretion, as well as improved glucose management. This not only extends our findings on a proinflammatory role of SIRT6 to chronic inflammation during obesity but also opens a plausible therapeutic window for treating inflammation and hyperglycemia during this disease. However, the latter has to be considered cautiously, and longer experiments need to be conducted in order to determine the duration of this protective effect. Since the majority of SIRT6, even during acute inflammation, is located in the nucleus, it is highly possible that during chronic SIRT6 deletion, with time the transcriptional control over proinflammatory genes regulated by SIRT6 becomes the dominant effect. Indeed, when we measured TNFα expression in SIRT6 KO MEFs, we found a dramatic decrease in TNFα expression in response to LPS, supporting this idea (Fig. S2). Finding the right time frame and dosage of SIRT6 inhibition may be key in order to correctly achieve anti-inflammatory and protective effects of SIRT6 inhibition, without affecting its major regulatory functions in the nucleus.

## Conclusions and perspectives

This work brings novel insight into how SIRT6 is actively regulated during acute inflammation, highlighting its role in the secretion of TNFα during inflammations. In addition, we show for the first time that SIRT6 controls TNFα secretion *in vivo* during LPS-dependent acute inflammation. We further extended these findings to chronic inflammation during obesity, suggesting that SIRT6-dependent TNFα secretion in macrophages play a key role in chronic systemic inflammation as well. However, we believe that further investigation needs to be conducted in order to clearly determine how macrophages, and maybe other immune cells, orchestrate a finely tuned SIRT6-dependent response to control inflammation.

## Experimental procedures

### General reagents and antibodies

All general reagents and chemicals were purchased from Sigma–Aldrich, unless otherwise specified. Supplements and media for cell culture were from Invitrogen. Antibodies were purchased from Cell Signaling Technology (anti-Sirt6, catalog no.: 12486; anti-p27, catalog no.: 3688; anti–histone H3, catalog no.: 4620; and antiubiquitin, catalog no.: 3936), Abcam (anti-TNFα, catalog no.: ab183218 and anti-SIRT6, catalog no.: ab191385), or Sigma–Aldrich (anti–beta actin, catalog no.: A5441 and anti–alpha tubulin, catalog no.: T6074). Antibodies for flow cytometry were purchased from Thermo Fisher Scientific (LIVE/DEAD Fixable Far Red Dead Cell Stain Kit; catalog no.: L10120), BioLegend (CD19-APCCy7, catalog no.: 115530; T-cell receptor beta (TCRβ)-APCCy7, catalog no.: 109220; Ly6G-APCCy7, catalog no.: 127624; CD11b-BV510, catalog no.: 101245; and ZOMBIE-AQUA, catalog no.: 423102), or Millipore (F4/80-PE, catalog no.: MABF1530).

### Cell growth and maintenance

MEFs were obtained from E13.5–E14.5 embryos following standard procedures. Raw 264.7 macrophages were obtained from American Type Culture Collection (ATCC-TIB-71). Cell growth and maintenance was performed in standard conditions in a humidified CO_2_ incubator. High-glucose Dulbecco's modified Eagle's medium (DMEM), supplemented with 10% fetal bovine serum (FBS), 2 mM glutamine, 10 mM Hepes, 10,000 U/ml penicillin, and 10,000 μg/ml streptomycin (complete DMEM) were used for both cell lines. Experiments in MEFs were performed between passages 2 and 5, whereas experiments in Raw 264.7 were performed between passages 15 and 22.

### *In vitro* assays and specific reagents

LPS from *Escherichia coli* serotype 0111:B4 (catalog no.: L3129; Sigma) was used at 200 ng/ml; MG132 (catalog no.: ab141003; abcam) was used at 10 μM; CHX (catalog no.: C6255; Sigma) was used at 1 μg/ml, and the SIRT6 inhibitor compound 1 was supplied by Chemspace (catalog no.: CSC000732205) and used at 200 μM in culture media. All the experiments were done in complete DMEM with 0.1% FBS. MG132, CHX, and the SIRT6 inhibitor were dissolved in DMSO. Control experiments for each treatment were done with DMSO alone. Final volumes in cell culture were always below 0.1%.

### Subcellular fractionation

Raw 264.7 cells were treated with LPS during 1 h. After, cells were washed with 5 ml of warm PBS (37 °C), harvested in 1 ml of PBS, and centrifuged (3 min, 500*g*). Pelleted cells were mechanically lysed in a hypotonic buffer (10 mM Hepes, 1 mM EDTA, and 0.1 mM EGTA containing 3% Triton X-100) containing a protease inhibitor cocktail (catalog no.: S8830; Sigma) with 10 strokes using a glass–pestle homogenizer, followed by 10 s of vigorous shaking. Proper cell lysis with simultaneous nuclear preservation was monitored by observation under microscope. The cell lysates were centrifuged at 13,000*g* for 10 min, yielding a supernatant (cytosolic fraction) and a pellet containing mostly nuclei and cytoplasmic debris. The nuclei-enriched pellet was further resuspended in radioimmunoprecipitation assay (25 mM Tris [pH 8.0], 150 mM NaCl, 1% NP-40, and 0.1% SDS) and subsequently sonicated in ice with five cycles of short bursts of 10 s, followed by intervals of 5 s to keep the suspension cool and avoid foaming. The supernatant obtained after a 10 min centrifugation (10,000*g*) represented our nuclear fraction.

### Fluorescent labeling of SIRT6, TNFα, and ER in macrophages

Raw 264.7 cells were incubated with LPS for 1 and 24 h (200 ng/ml) in order to induce activation. ER tracker (catalog no.: E34250; Invitrogen) was used at 1 μM final concentration in the cell medium and was incubated 30 min before fixation. Peritoneal cells were centrifuged in cytospin after peritoneal lavage. In all cases, cells were fixed with 4% paraformaldehyde for 15 min at room temperature (RT), permeabilized, and blocked (1% bovine serum albumin, 0.1% saponin, 150 mM glycine, and 5% FBS in PBS) for 1 h at RT. Primary antibodies against SIRT6 and TNFα were prepared in antibody dilution buffer (1% bovine serum albumin, 0.1% saponin, and 150 mM glycine in PBS) and incubated overnight at 4 °C. Alexa-conjugated secondary antibodies were incubated for 1 h. Nuclei were stained using 4′,6-diamidino-2-phenylindole (catalog no.: D9542; Sigma) and actin filaments with phalloidin (catalog no.: A22283; Invitrogen). Samples were mounted in Prolong Gold Antifade Reagent (catalog no.: P10144; Invitrogen), and images were acquired using a Zeiss LSM 880 spectral confocal laser scanning microscope, using a 60× oil-immersion objective (numerical aperture of 1.45). Images were processed using ImageJ (Wayne Rasband, National Institutes of Health).

### Animal handling and experiments

All mice used in this study (male C57BL/6) were bred and maintained at the Institut Pasteur Montevideo Animal facility (UBAL). The experimental protocol was approved by the Institutional Animal Care and Use Committee of the Institut Pasteur Montevideo (CEUA; protocol numbers 70153-000839-17, 003-19, and 006-19). All the studies described were performed according to the methods approved in the protocol and following all international guidelines and legal regulations. Mice received standard chow water *ad libitum* or WD (catalog no.: 5TJN-1810842; TestDiet) and water containing glucose and fructose (18.9 and 23.1 g/l, respectively).

### LPS challenge and survival mice studies

Adult mice (4–5 months) were injected intraperitoneally with compound 1 (SIRT6 inhibitor, 30 mg/kg) or vehicle (DMSO). After 2 h, mice were injected with 10 mg/kg of LPS (for TNFα release) or 20 mg/kg LPS (for survival experiments) in PBS. Survival was checked every 12 h for 4 days. For each animal, a severity score was calculated according to clinical parameters, such as weight, physical appearance, and motor activity. Those animals above a score of 5 were euthanized. Survivors at the end of the experiment were euthanized by cervical dislocation.

### Thioglycollate-elicited macrophages in peritoneal cavity and LPS treatment

Mice were injected intraperitoneally with 800 μl of 4% (w/v) of brewer thioglycollate medium (Sigma). At 72 h, mice were injected with 10 mg/kg of LPS in PBS. After 2 h, the peritoneal lavage was performed, injecting 3 ml of RPMI medium + 0.2% FBS into the peritoneal cavity and recovering this volume to obtain the cells to be analyzed by immunofluorescence.

### BMDM generation and LPS treatment

BMDMs were isolated from 20-week-old male mice. To obtain bone marrow cells, the femur and tibia were flushed with complete DMEM supplemented with 10% FBS. Cells were plated into 100 mm Petri dish with complete DMEM supplemented with 20 ng/ml recombinant mouse macrophage colony-stimulating factor protein (catalog no.: ab129146; Abcam). Four days after seeding, cells were subcultured in the same medium and grown on glass coverslips for 3 days until differentiated into BMDM. On day 8, BMDMs were treated for 3 h with LPS (200 ng/ml) and later fixed in 4% paraformaldehyde for 15 min for immunofluorescence analysis. To confirm Sirt6 deletion, BMDM from Sirt6^loxp/loxp^; Cre+ were obtained, and 4-OH-Tamoxifen (2 μM) was added to cell medium 6 days after seeding. Twenty-four hours later, the cells were processed for Western blot and quantitative PCR.

### Macrophage-specific Sirt6 KO mice generation and treatments

Mice carrying a SIRT6 conditional allele (017334; Jackson Laboratories) were crossed with a transgenic mice expressing the tamoxifen-inducible MerCreMer fusion protein under control of the macrophage-specific mouse *Csf1r* (Tg[Csf1r-Mer-iCre-Mer]1Jwp; catalog no.: 019098; Jackson Laboratories) in order to conditionally delete Sirt6 in macrophages. Transgenic mice used were previously backcrossed into C57BL/6 J for more than 10 generations in order to reach a homogeneous background. For activation of MerCreMer, 50 mg/kg of tamoxifen (catalog no.: T5648; Sigma) in corn oil (catalog no.: C8267; Sigma) were administered to 5-month-old mice once a day for seven consecutive days. Tamoxifen-treated Sirt6^loxp/loxp^;Cre− mice were used as controls. For DIO studies, adult mice (3 months) were monitored for body weight and basal glycemia before and after WD administration for 10 weeks. In weeks 8 and 9, tamoxifen injections (50 mg/kg) were done for three consecutive days. At week 10 of WD, mice were euthanized with an excess of ketamine/xylazine solution followed by cervical dislocation. Sample and blood collection were taken for posterior analyses.

### Glycemia determination

Basal glycemia was controlled in 12 h-fasted mice on standard diet or WD. Plasma glucose concentrations were measured in blood from the tail using a hand-held glucometer (Accu-Chek; Roche).

### Hemogram and biochemical parameters

Blood analyses were done with 30 μl of blood in Hemocytometer Mindray BC-5000Vet. Kidney and Liver parameters were measured with 100 μl of blood in MNCHIP (PointCare V2).

### Western blotting

Cells were pelleted (5 min, 500*g*) and lysed using radioimmunoprecipitation assay buffer (in a volume ratio of 1:10) supplemented with 5 mM NaF, 5 mM nicotinamide, 50 mM β-glycerophosphate, 1 μM trichostatin A (catalog no.: 647925; Sigma), a protease inhibitor cocktail, and then sonicated by means of five cycles of 10 s each followed by intervals of 5 s. Homogenates were incubated during 20 to 30 min at 4 °C under constant agitation and then centrifuged at 10,000*g* during 10 min. Protein concentrations in the supernatants were determined using the Bradford protein assay reagent. Samples were resuspended in Laemmli 5×, separated in SDS-PAGE gels, and transferred to polyvinylidene fluoride membranes. After blocking (with Tris-buffered saline containing 0.2% Tween-20 and 5% nonfat milk), the membranes were incubated overnight with the appropriate antibodies. Secondary antibodies were incubated 1 h and detected using SuperSignal West Pico Chemiluminescent Kit (catalog no.: 34080; Pierce). Results were processed by densitometry analysis with ImageJ (Rasband W.S.; National Institutes of Health).

### RNA isolation and quantitative PCR

Cells were homogenized in TRIzol reagent for RNA extraction according to the manufacturer's protocol (catalog no.: 15596026; Invitrogen). DNase I treatment was used to eliminate genomic DNA contamination (catalog no.: 04716728001; Roche). Reverse transcription (1 μg) was done using SuperScript II RT (catalog no.: 18064-014; Invitrogen), and quantitative RT–PCR was performed using Fast SYBR Green Mix (catalog no.: 04913850001; Roche) in QuantStudio3 thermocycler (Applied Biosystems). Gene expression analysis was calculated using the ΔΔCt method with β-actin as the housekeeping gene. Expression was calculated as fold increase over control condition. Primers were synthesized by Integrated DNA Technology and are listed in Table S2.

### TNFα detection in Raw 264.7 supernatant, serum, and peritoneal lavage of mice

Raw 264.7 cells were plated 24 h before incubation with compound 1 (SIRT6 inhibitor, 200 μM) in DMEM and 0.1% FBS. After 1 h of compound 1 incubation, cells were exposed to LPS (200 ng/ml) for an additional hour. The presence of TNFα in the culture medium was determined by ELISA (catalog no.: 555268; BD OptEIA). TNFα levels were also measured in mice serum and peritoneal lavage after LPS challenge and tamoxifen injections.

### Flow cytometry analysis of SIRT6 expression in macrophages in the peritoneal cavity

Flow cytometry analysis was performed to evaluate SIRT6 expression in peritoneal cells. The peritoneal washes were carried out by injecting 3 ml of RPMI medium + 0.2% FBS and recovering this volume to obtain the cells to be analyzed. Cell surface staining was performed for 30 min at 4 °C using the antibodies against murine CD19 (1:400 dilution), CD11b (1:300 dilution), and F4/80 (1:200 dilution); in cases where the peritoneal cavity was treated with inflammatory stimuli, anti-TCRβ (1:400 dilution) and anti-Ly6G (1:400 dilution) antibodies were also added. For SIRT6 intracellular labeling, cells were fixed and permeabilized overnight at 4 °C. Cells were stained with anti-SIRT6 antibody (1:200 dilution) for 30 min and then with a goat anti-rabbit secondary antibody (1:200 dilution) for 1 h. Fluorescence Minus One controls were added, corresponding to anti-SIRT6 or the secondary antibody. The acquisition of the samples was performed using Attune NxT cytometer (Thermo Fisher Scientific), and the analysis was performed with FlowJo, version X.0.7 software. Dead cells were excluded using Zombie or live/dead fixable dead cell markers. Macrophage populations within the peritoneal cells were defined as CD19^−^CD11b^+^F4/80^lo^ (recruited macrophages) and CD19^−^CD11b^+^F4/80^hi^ (resident macrophages); under inflammatory conditions, the same populations were identified as CD19^−^TCRβ^−^Ly6G^−^CD11b^+^F4/80^lo^ (recruited macrophages) and CD19^−^TCRβ^−^Ly6G^−^CD11b^+^F4/80^hi^ (resident macrophages). When B cells were analyzed, they were defined as CD19^+^ cells. SIRT6 expression in all mentioned populations was expressed as the percentage of SIRT6-positive cells and SIRT6 geometric mean fluorescence intensity in SIRT6-positive cells, as a measure of SIRT6 expression levels within this population. The expression thresholds for each group and experiment were determined with the help of the Fluorescence Minus One controls.

### Statistical analysis

All data are presented as mean ± SD. D'Agostino–Pearson analyses were performed to confirm normal distributions. ROUT method was used to identify outliers. Unpaired *t* test was used to compare two independent groups. In multiple comparisons, ANOVA followed by Tukey's post hoc test was used. Under non-normal distribution, data were expressed as median ± 95% confidence interval. Mann–Whitney *U* test was used to compare two independent samples (groups). Kruskal–Wallis test was used for multiple comparisons in cases where sample distributions were not normal, followed by Dunn's post hoc test. For comparisons of proportions, Fisher's exact test was used. In all cases, *p* < 0.05 was considered to be significant. Calculations were done using GraphPad Prism 7.0 (GraphPad Software, Inc).

## Data availability

All the data presented are contained within the article. Any additional information, reagents, or mice models will be shared upon request to the lead contact, Carlos Escande, PhD (escande@pasteur.edu.uy).

## Supporting information

This article contains supporting information.

## Conflict of interest

The authors declare that they have no conflicts of interest with the contents of this article.

## Figures and Tables

**Supplemental Figure S2:**