# Supplementary material for: SIRT6 stabilization and cytoplasmic localization in macrophages regulates acute and chronic inflammation in mice
Source: J Biol Chem. 2022 Feb 9;298(3):101711. doi: 10.1016/j.jbc.2022.101711 (PMC8913316; doi:10.1016/j.jbc.2022.101711)
Supplement: Supplemental Table S1 [file mmc1.docx]

| **Liver Panel** | | | |
| --- | --- | --- | --- |
|  | Sirt6loxP/loxP;Cre-   (n=5)  Mean ± SEM | Sirt6loxP/loxP;Cre+ (n=5)  Mean ± SEM | P value |
| TP-Total Protein (g/L) | 43,8 ± 1,4 | 44,7 ± 1,2 | 0,63 |
| ALB-Albumin (g/L) | 23,2 ± 2,5 | 25,7 ± 1,7 | 0,61 |
| GLO-Globulin (g/L) | 20,6 ± 2,8 | 19,3 ± 1,7 | 0,62 |
| ALB/GLO ratio | 1,5 ± 0,4 | 1,48 ± 0,2 | 0,98 |
| TBIL-Total Bilirrubin (uM) | 3,4 ± 0,4 | 4,17 ± 0,3 | 0,19 |
| ALT-Alanine Aminotransferase (U/L) | 35,2 ± 4,9 | 20,88 ± 52,3 | 0,01 (**) |
| AST-Aspartate Aminotransnferase (U/L) | 238,3 ± 50,8 | 180,9 ± 32,5 | 0,33 |
| GGT- Gamma-glutamyl transpeptidase (U/L) | 0,78 ± 0,2 | 1,22 ± 0,2 | 0,14 |
| **Kidney** **Panel** | | | |
|  | Sirt6loxP/loxP;Cre-   (n=5)  Mean ± SEM | Sirt6loxP/loxP;Cre+ (n=5)  Mean ± SEM | P value |
| BUN-Blood Urea Nitrogen (mM) | 6,8 ± 0,4 | 8,1 ± 0,4 | 0,08 |
| CRE-Creatinine (µM) | 66,9 ± 6,2 | 43,5 ± 4,8 | 0,01 (**) |
| BUN/Creat ratio. | 26,2 ± 2,4 | 46,8 ± 5,4 | 0,01 (**) |
