# Supplementary material for: SIRT6 stabilization and cytoplasmic localization in macrophages regulates acute and chronic inflammation in mice
Source: J Biol Chem. 2022 Feb 9;298(3):101711. doi: 10.1016/j.jbc.2022.101711 (PMC8913316; doi:10.1016/j.jbc.2022.101711)
Supplement: Supplemental Table S2 [file mmc2.docx]

| ***Primers Mouse*** | ***Sequence 5’---3’*** |
| --- | --- |
| **Mm_SIRT6_vo_F** | **ATGTCGGTGAATTATGCAGCA** |
| **Mm_SIRT6_vo_R** | **GCTGGAGGACTGCCACATTA** |
| **Mm_SIRT6_v1_F** | **ATCTTCGACCCACCAGAGG** |
| **Mm_SIRT6_v1_R** | **GCATTCTCGAAGGTGGTGTC** |
| **Mm_Actin_F** | **AGCCATGTACGTAGCCATCC** |
| **Mm_Actin_R** | **GCTGTGGTGGTGAAGCTGTA** |
| **Mm_TNFα_F** | **CCAGACCCTCACACTCAGATC** |
| **Mm_TNFα_R** | **CACTTGGTGGTTTGCTACGAC** |
| **Mm_IL6_F** | **CCACAGATACAAAGAAATGAT** |
| **Mm_IL6_R** | **ACTCCAGAAGACCAGAGGAAT** |
