# Supplementary material for: SIRT6 stabilization and cytoplasmic localization in macrophages regulates acute and chronic inflammation in mice
Source: J Biol Chem. 2022 Feb 9;298(3):101711. doi: 10.1016/j.jbc.2022.101711 (PMC8913316; doi:10.1016/j.jbc.2022.101711)
Supplement: Supplemental Figures and Tables Legends [file mmc3.pdf]

SIRT6 stabilization and cytoplasmic localization in macrophages regulates acute and chronic inflammation in mice

Running title: SIRT6 regulates TNFα secretion in macrophages in vivo

Mariana Bresque[1,2]; Karina Cal[1,3]; Valentina Pérez-Torrado[1,5,7]; Laura Colman[1,6]; Jorge Rodríguez-Duarte[2]; Cecilia Vilaseca[4]; Leonardo Santos[1]; María Pía Garat[1]; Santiago Ruiz[1]; Frances Evans[8]; Rosina Dapueto[1,2]; Paola Contreras[1,4]; Aldo Calliari[1,3]; Carlos Escande[1*]

Supporting Information

Supplementary Figure Legends

Supplementary figure 1. SIRT6 is up-regulated in response to LPS in MEFs. **A**. Representative Western blot of SIRT6 in MEF cells exposed to LPS (200ng/ml) for 1, 6 and 24h. **B**. Densitometry readings of SIRT6 relative to tubulin of WB shown in A and its replicates. **C**. *Sirt6* mRNA levels in MEF cells exposed to LPS for 1, 6 and 24h. **D-F**. Densitometry of SIRT6 levels relative to control samples in MEF cells exposed to LPS (200ng/ml) (**D**), MG132 (10μM) (**E**), or CHX (1μg/ml) (**F**) for 1h. **G.** Representative WB of SIRT6 in MEF cells exposed to MG132 or CHX for 1h measured in E-F. **H**. Representative WB of SIRT6 in MEF cells exposed to MG132 and/or LPS for 1h. Ubiquitin is shown as a control for MG132 treatment. **I**. Densitometry readings of SIRT6 WB shown in H and its replicates. Data represent mean ± SD, *p<0,05, **p<0,01, ***p<0,001, ****p<0,0001. All experiments were repeated at least 3 or 4 times.

Supplementary figure 2. **A.** *Antibody control in siSIRT6 MEF Cells.* Representative confocal images of IF of MEF cells silenced for SIRT6 and controls, stained with DAPI (gray) and SIRT6 (green). White arrows indicate nuclear signal for SIRT6 and red arrows indicate cytoplasmic SIRT6 signal. White bar represents 10 µm. **B**. *Tnfα* mRNA levels in MEF WT and SIRT6 KO cells exposed to LPS (200ng/ml) for 1h. **C**. Released TNFα levels measured by ELISA in the supernatant of MEF cells exposed to LPS (200ng/ml) for 1h and treated with the SIRT6 inhibitor compound 1. Data represent mean ± SD, *p<0,05, **p<0,01, ***p<0,001, ****p<0,0001. **D-E.** Cytoplasmic SIRT6 up-regulation in response to LPS (200 ng/ml) treatment (3 hours) in bone marrow derived primary macrophages (BMDM). **D.** Representative

immunofluorescence. White arrows point to the cytoplasmic SIRT6 signal. **E.** Quantitation of Mean Fluorescence intensity in the cytoplasm under the different experimental conditions. Data represent mean ± SD ***p<0,001.

Supplementary Table I- Liver and Kidney panel in SIRT6$^{lox/loxp}$;Cre- and SIRT6$^{lox/loxp}$; Cre+ on Western diet and treated with Tamoxifen.

Supplementary Table II – qPCR sequences primer list
